# Using transcranial direct current stimulation (tDCS) to selectively modulate the face inversion effect and N170 event-related potentials

**DOI:** 10.1177/03010066231215909

**Published:** 2023-11-28

**Authors:** Ciro Civile, Emika Waguri, I.P.L. McLaren

**Affiliations:** 3286 University of Exeter, UK

**Keywords:** face inversion effect, perceptual learning, tDCS, N170

## Abstract

We report a large study (*n* = 72) using combined transcranial direct current stimulation-electroencephalography (tDCS-EEG) to investigate the modulation of perceptual learning indexed by the face inversion effect. Participants were engaged with an old/new recognition task involving intermixed upright and inverted, normal and Thatcherized faces. The accuracy results showed anodal tDCS delivered at the Fp3 scalp area (cathode/reference electrode placed at Fp2) increased the behavioural inversion effect for normal faces versus sham/control and this covaried with a modulation of the N170 event-related potential component. A reduced inversion effect for normal faces was found on the N170 latency and amplitude versus sham/control, extending recent work that combined tDCS and EEG in circumstances where the behavioural face inversion effect was reduced. Our results advance understanding of the neural mechanisms responsible for perceptual learning by revealing a dissociation between the N170 amplitude and latency in response to the tDCS-induced modulation of the face inversion effect. The behavioural modulation of the inversion effect tracks the modulation of the N170 amplitudes, albeit it is negatively correlated (i.e., reduced inversion effect—larger N170 amplitude inversion effect, increased inversion effect—reduced N170 amplitude inversion effect). For the N170 latencies, the inversion effect is reduced by the tDCS protocol we use irrespective of any modulation of the behavioural inversion effect.

The face inversion effect is one of the most reliable and replicated cognitive phenomena in the psychological literature. Several published studies have demonstrated that when individuals are presented with real life or standardized faces turned upside down, their recognition performance is severely disrupted compared to when the same faces are presented in their usual upright orientation (Civile, McLaren, et al., 2014; Civile, McLaren et al., 2016; Diamond & Carey, 1986; Farah et al., 1995; Valentine, 1988; Yin, 1969). In the last decade some of the most significant advances into the mechanisms of face recognition skills have derived from research using transcranial direct current stimulation (tDCS) to modulate the face inversion effect. In this paper, we will combine this technique with electroencephalography (EEG)/event related potentials (ERP) analysis to characterize the mechanisms responsible for perceptual learning and face recognition.

## Background

When it was first discovered the inversion effect was used to provide evidence in support of the specificity account of face recognition (Farah et al., 1995; Yin, 1969). However, this was later challenged by studies that found how a robust inversion effect as pronounced as that for faces, can be obtained from sets of stimuli that participants are familiar with (e.g., dog images for dog breeders, Diamond & Carey, 1986; Gauthier & Tarr, 1997; checkerboards, McLaren, 1997, McLaren & Civile, 2011) suggesting how expertise rather than face specificity is a key factor at the basis of the inversion effect. In 2014, by adopting an old/new recognition task typically used to study the face inversion effect, Civile, Zhao et al. (2014) showed how a robust inversion effect is found for prototype-defined categories of non-mono-orientated (i.e., no predefined orientation) artificial stimuli (i.e., checkerboards) that participants had been pre-exposed to. Participants first performed a categorization learning task, where they were asked to categorize two sets of prototype-defined checkerboard stimuli. Following, they were asked to first memorize a set of upright (same orientation as in the categorization task) and inverted (rotated by 180 degrees) checkerboards from one of the categories seen previously (i.e., familiar) in addition to checkerboards from a novel category not seen previously. In the final phase, participants performed an old/new recognition task which revealed a robust inversion effect for checkerboards drawn from the familiar category (i.e., higher recognition performance for upright vs. inverted checkerboards).

The results from Civile, Zhao, et al. (2014) were interpreted using the MKM model which is based on the modulation of salience by error to produce perceptual learning (McLaren et al., 2012; McLaren et al., 1989; McLaren & Mackintosh, 2000). According to this model, the characteristic/common features of a stimulus are well predicted by other features (i.e., low error) and so they would tend to have lower salience (i.e., activation), whereas the features that are novel and so not predicted by other features, would tend to have higher salience. Applied to explain the checkerboard inversion effect, during the pre-exposure phase (the categorization task), participants associate the prototypical features shared by the checkerboards with the correct category membership. Each of these common features would lose their salience after being presented on every trial and would become strongly predicted by other features present. This would lead to perceptual learning because the features unique to each checkerboard would still have high salience due to less exposure and lower predictability. When asked to recognize the checkerboards it would be easier for the participants to do so because the salience of the common features is now low, whereas that of the unique features is still high. This mechanism of feature salience modulation regards mainly upright checkerboards because the participants would have less experience in seeing inverted ones and so performance on these is not aided by any significant amount of perceptual learning.

Civile, Verbruggen, et al. (2016) adapted a tDCS procedure previously used in the learning and categorization literature (Ambrus et al., 2011; McLaren et al., 2016) to the Civile, Zhao, et al. (2014)'s checkerboard inversion effect paradigm. Hence, a bilateral bipolar-non-balanced montage with one electrode (anode) placed over the target stimulation area (Fp3) and the other electrode (cathode/reference) on the opposite supraorbital area (Fp2) was used to investigate how anodal tDCS (targeting the dorsolateral prefrontal cortex) would influence the checkerboard inversion effect. The results from the sham/control tDCS group confirmed the robust inversion effect for checkerboards from a familiar category. The results from the anodal group revealed a significantly reduced inversion effect compared to that found in the sham group. Furthermore, recognition performance for upright familiar checkerboards was significantly reduced by the anodal tDCS versus that in the sham group. The same tDCS procedure was later applied to the face inversion effect revealing in this case as well a significantly reduced inversion effect in the anodal versus sham group was found, and this was mainly due to a disrupted recognition performance for upright faces in the anodal group (Civile, Cooke, et al., 2020; Civile, McLaren, et al., 2018; Civile, Obhi et al., 2019; Civile, McLaren et al., 2020; Civile, Quaglia et al., 2021); Civile, Waguri, et al., 2020. It is also important to highlight that in addition to the sham control, authors have also conducted *active control* studies which revealed how targeting different scalp areas with the tDCS does not influence the inversion effect (Civile, McLaren, et al., 2018; Civile, McLaren, et al., 2021).

Based on the MKM model, when the anodal tDCS procedure is used, the anodal stimulation disables the mechanism for salience modulation based on error by strongly inhibiting this process, which means that instead of prior pre-exposure to a prototype-defined category enhancing the discriminability of the exemplars taken from that category, it now, enhances generalization between them. Features common to exemplars would be more prominent because they co-activate one another, whereas unique features found in one or very few exemplars would have low salience as they do not receive additional activation. It is this change in perceptual learning that causes the reduction in the inversion effect because it reduces subjects’ ability to discriminate between different upright stimuli, essentially making them look more “similar” to one another. This explanation was confirmed when the same tDCS procedure was applied to a prototype-distortion task where increased salience of the prototypical features would lead to improved task performance (McLaren et al., 2016).

Further evidence on how tDCS affects perceptual learning comes from recent work where the same tDCS procedure, was used to *enhance* the inversion effect in circumstances where harmful generalization affected performance for upright faces. The experiment in question employed normal upright and inverted faces presented with upright and inverted Thatcherized faces (Civile, Cooke, et al., 2020). Generalization between faces depends on how similar they are to one another. If they are similar, then there is lots of generalization and the recognition task (which involves telling seen from unseen faces) will be difficult. If they are not, then less generalization ensues, and the task will be easier. Typically, there will be some features that are common to most faces and promote generalization. Others might be shared by a few faces, yet others are unique to a face, and these features are obviously very important for correct discrimination between exemplars. Thatcherized faces are a set of specially manipulated faces where the eyes and mouth have been rotated by 180° while the rest of the face remains upright. The result is a face that, when it is presented upright, the rotated eyes and mouth stand out as rather “odd” and salient, however, if inverted, the manipulated features are not so easily apparent (Civile et al., 2012; Civile et al., 2011; Donnelly & Hadwin, 2003; Leder et al., 2001; Lewis, 2001; Lewis & Johnston, 1997; Murray et al., 2000; Searcy & Bartlett, 1996; Thompson, 1980).

Importantly, from a perceptual learning perspective, Thatcherized faces have the same features as normal faces, but some of them have now been rotated, and so are super-salient (because not predicted by other features) inducing generalization to any given normal face. In addition, there will be other features that have not been manipulated, in Thatcherized faces that are shared with normal faces, which would however, also have higher salience than usual, because they are not predicted quite as well as in the case of normal faces, as some of the usual predictors are now incorrect. This will also increase generalization to normal faces, and so make discrimination harder. Presenting Thatcherized faces with normal faces, then, will make performance with the normal faces worse, and because this will affect the upright faces more than the inverted ones, the inversion effect will be reduced (Civile, Cooke, et al., 2020). However, there will not be a symmetric effect of generalization from normal to Thatcherized faces, because the salient features in normal faces are simply not as salient as the super-salient ones in Thatcherized faces and will not tend to occur in many faces, Thatcherized or otherwise, because they are the distinctive features of a face and hence rare.

Through a series of experiments where participants were engaged in an old/new recognition task involving upright and inverted normal and Thatcherized faces presented intermixed, the authors provided evidence in support of the tDCS procedure able to reduce the harmful effect of generalization from Thatcherized to normal faces thus leading to an increased inversion effect for normal faces compared to sham mainly based on recognition performance for upright normal faces being enhanced by the anodal stimulation. In a further extension the authors showed how within the same experiment the anodal tDCS significantly reduced the inversion effect versus sham when normal faces were presented with other normal faces (male and female faces). But when normal faces were presented with Thatcherized faces (male normal and Thatcherized faces) the inversion effect for normal faces was significantly increased by the same anodal tDCS procedure (Civile, Cooke, et al., 2020). Overall, there is now good evidence in the literature showing that when anodal tDCS is delivered at Fp3 site (with the cathode/reference electrode placed at Fp2), the inversion effect for normal faces can be either reduced or enhanced depending on the stimuli they are intermixed with. Recently, the effects of tDCS on the inversion effect expected on the basis of our analysis for normal faces when presented in isolation or when presented intermixed with Thatcherized faces, were confirmed by simulation work conducted using a MatLab implementation of the MKM model (Civile et al., 2023).

In the current paper, we will combine tDCS and EEG/ERPs to characterize the electrophysiological correlates of the inversion effect for normal faces when presented intermixed with Thatcherized faces. The most studied ERP component associated with face stimuli is the N170 which is a negative-polarity deflection (peak) maximal at 130 to 210 ms after the stimulus onset and it is found to be largest at parietal-occipital regions (Bentin et al., 1996; Carmel & Bentin, 2002; Rossion & Corentin, 2011). Several studies found that normal inverted faces elicited a delayed N170 latency compared to upright faces which also resulted in an enhancement of the N170 amplitude. Thus, a delayed and larger N170 for inverted versus upright regular faces is one index of an inversion effect (Civile, Elchlepp, et al., 2018; Eimer, 2011; Jacques & Rossion, 2007; Marzi & Viggiano, 2007; Righart & de Gelder, 2006). Studies in support of the expertise account of the inversion effect have also revealed a delayed and larger N170 for inverted versus upright familiar artificial stimuli (e.g., Greebles, checkerboards) suggesting how modulation of the N170 could be associated with disruption of the expertise (Busey & Vanderkolk, 2005; Civile, Zhao, et al., 2014; Rossion et al., 2002).

So far in the literature, only one study has investigated the tDCS effects on ERPs in response to the face inversion effect. Adopting combined tDCS and EEG simultaneously, Civile, Waguri, et al. (2020) characterized the effects of anodal tDCS at Fp3 with the cathode/reference at Fp2, on the N170 component in response to upright and inverted normal faces presented alone. Hence, the tDCS procedure and behavioural task were the same as those previously used in the literature (e.g., Civile, McLaren, et al., 2018), with the addition of EEG. The behavioural accuracy results confirmed that anodal tDCS reduced the inversion effect compared to sham by means of impaired recognition performance for upright faces. ERP results from the P08 channel (selected because it has shown bigger effects on the N170 in response to faces, e.g., Civile, McLaren, et al., 2018; Navajas et al., 2013; Prieto et al., 2011; Rossion & Jacques, 2008) revealed a significantly reduced face inversion effect (i.e., less delay between inverted vs. upright faces) on the N170 latency in the anodal group versus that found in the sham group. Contrastingly, on the N170 amplitude the face inversion effect was increased (i.e., larger amplitude for inverted vs. upright faces) by the anodal stimulation versus that found in the sham group—an entirely novel effect. These effects were found to be consistent from brain responses recorded during the study phase (i.e., the learning phase) and the recognition phase (Civile, Waguri, et al., 2020).

In the current study, we extend Civile, Waguri, et al. (2020)'s work, by investigating the effects of the same tDCS procedure on the N170 when normal faces are presented intermixed with Thatcherized faces (*n* = 72). Hence, by applying tDCS and EEG simultaneously on the same recognition task with normal and Thatcherized faces as used in previous studies (Civile, Cooke, et al., 2020), we aim to characterize for the first time in the literature, the tDCS effects on the N170 in circumstances when the inversion effect for normal faces is enhanced, rather than reduced, by the tDCS procedure. This would help us understand how the electrophysiological components of the face inversion effect, that is, the N170 amplitude and latency, correlate both with the behavioural effect and with one another, potentially revealing different underlying mechanisms involved in this robust phenomenon. Importantly, the conditions used in this study would also be critical for our investigation of the perceptual learning processes involved in behavioural modulation of the face inversion effect. The results from this study will advance our understanding of the neural mechanisms controlling and modulating perceptual learning and feed into our simulation work modelling these effects (e.g., Civile et al., 2023).

## Method

### Subjects

In total, 72 naïve (right-handed) subjects (20 male, 52 female; mean age = 20.4 years, age range = 18–30) took part in the experiment. Subjects were randomly assigned to either sham or anodal tDCS groups (36 in each group). All the subjects were students from the University of Exeter. All methods were performed in accordance with the relevant guidelines and regulations approved by the CLES Psychology Research Ethics Committee at the University of Exeter. Informed consent was obtained from all subjects. The sample size was determined based on previous studies that have used the same old/new recognition paradigm, stimuli counterbalance, tDCS procedure and double-blind between-subject design (Civile, Cooke, et al., 2020; Civile, McLaren, et al., 2018; Civile et al., 2019; Civile, Quaglia, et al., 2021; Civile, Waguri, et al., 2020).

### Materials

**Figure 1.**
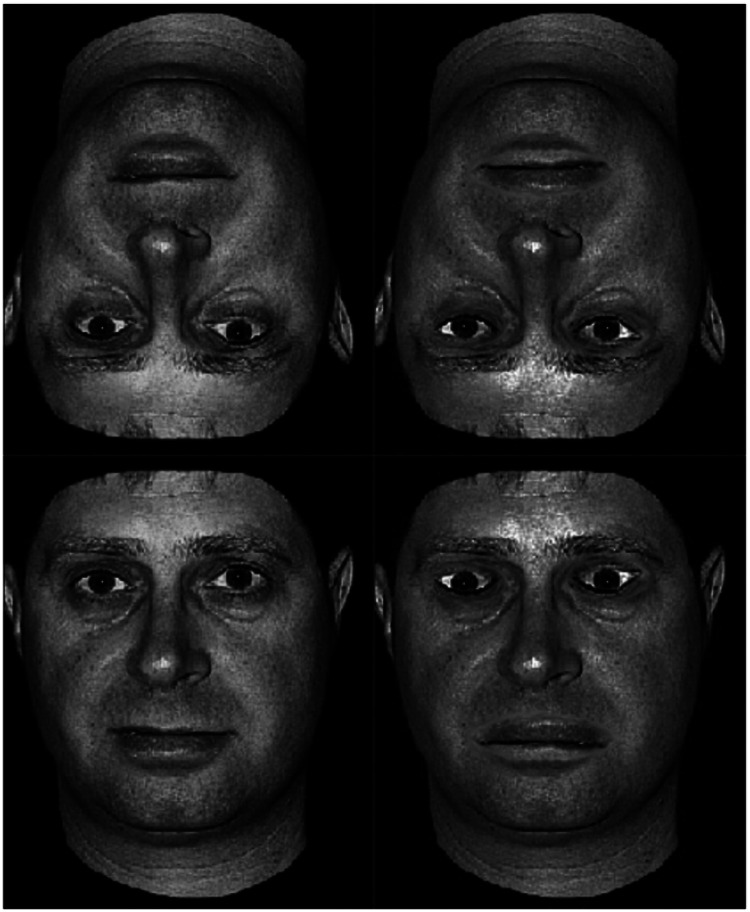
An example of the four stimulus’ conditions used to demonstrate the Thatcher illusion. The top half of the figure compares directly the same inverted face either as normal (left side) or Thatcherized (right side). The bottom half of the figure compares the same normal and Thatcherized face this time presented in its upright orientation. In the upright orientation it is noticeable how the Thatcherized version of the face has been manipulated at the eyes and the mouth as in the original Thatcher illusion (Thompson, 1980).

The experiment used the same set of 128 faces previously used in Civile, Cooke, et al. (2020). The original images were selected from the Psychological Image Collection at Stirling open access database (https://pics.stir.ac.uk). All faces were standardized using a grayscale colour on a black background. For all faces we cropped the hair so to avoid the possibility that subjects would find it easier to remember the faces based on the different hairstyles. The faces were prepared in four different versions, that is, normal upright, normal inverted, Thatcherized upright and Thatcherized inverted (see Figure 1). The Thatcherized faces were produced by first rotating the mouth and each of the eyes individually by 180 degrees in the upright faces. The area around the mouth and the eyes was then smoothed to blend in colour and luminosity with the rest of the faces. The same upright Thatcherized faces were copied and used to create the inverted Thatcherized faces to make sure that they matched in the manipulations applied (Civile, Cooke, et al., 2020; Civile et al., 2012; Civile et al., 2011). The stimuli, whose dimensions were 5.63 cm × 7.84 cm, were presented at resolution of 1280 × 960 pixels.

### The Behavioural Task

**Figure 2.**
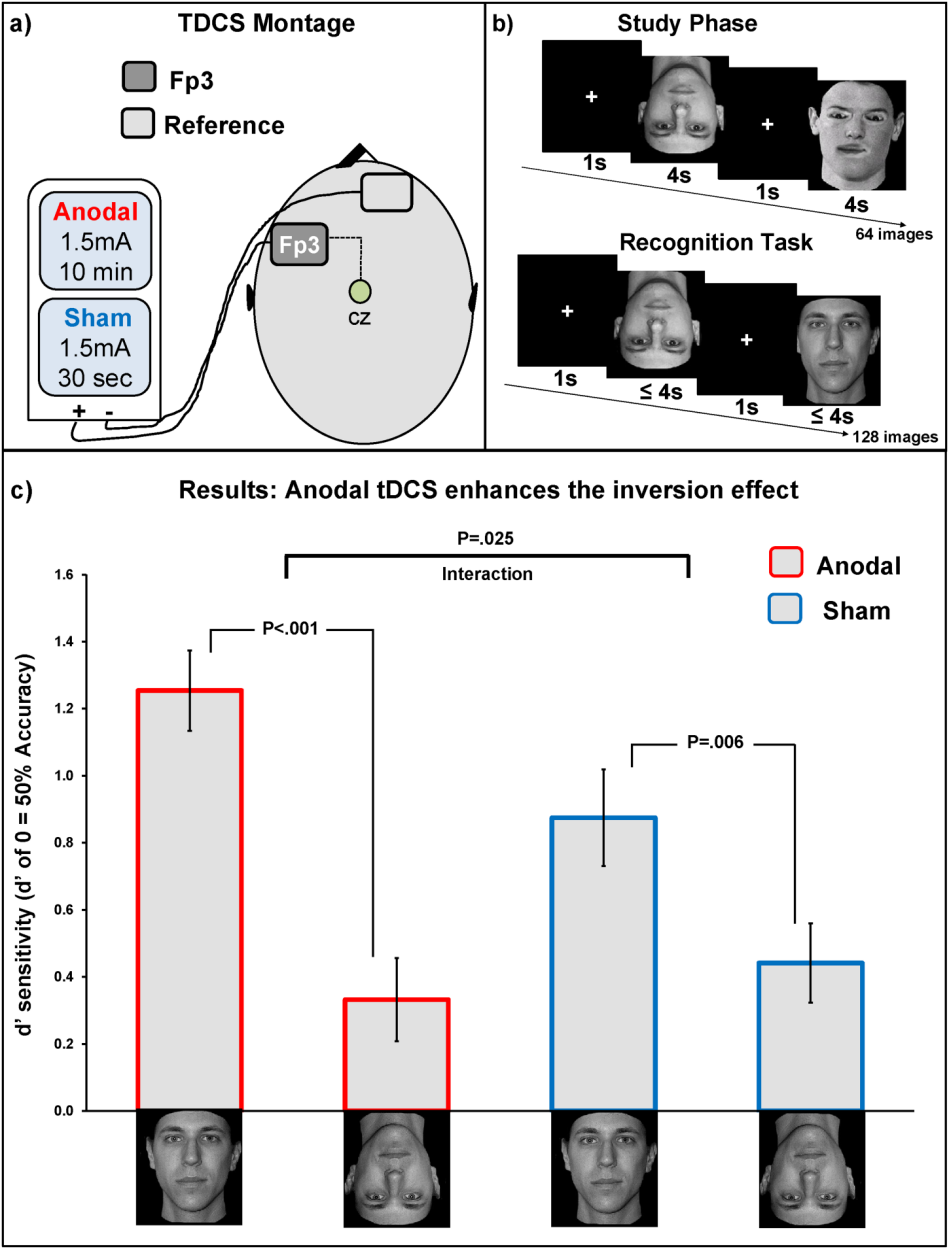
*Panel a* illustrates the tDCS montage used. This montage was the same as that used in previous studies on perceptual learning and the inversion effect. *Panel b* illustrates a schematic representation of the old/new recognition task used in which was the same task as that used by Civile, Cooke, et al. (2020). *Panel c* shows the behavioural for the normal faces only. The *x*-axis represents the normal upright and inverted faces across the two tDCS conditions (anodal, sham). The *y*-axis represents *d’* sensitivity measure. Error bars are s.e.m.

The experiment used the same behavioural task as that used in Civile, Cooke, et al. (2020) consisting of a “study phase” and an “old/new recognition phase” (Figure 2b). In the study phase, subjects were shown 32 normal faces (16 upright and 16 inverted) and 32 Thatcherized faces (16 upright and 16 inverted) presented one at a time in random order. In each trial of the study phase participants saw a fixation cross in the centre of the screen, for 1 s, then a face image was presented for 4 s before moving on to the next trial. After all the 64 face stimuli had been presented, the program displayed a set of instructions, explaining the recognition task. In the recognition phase, 64 normal and Thatcherized faces (half upright and half inverted) were showed intermixed with the 64 faces seen in the study phase, and all 128 stimuli were presented one at a time in random order. For a given subject, each face stimulus only appeared in one orientation during the experiment. The faces were each shown for 4 s (preceded by a 1 s fixation cross) and subjects pressed the ‘.’ key if they recognized the face as having been shown in the study phase or pressed ‘x’ if they did not (the keys were counterbalanced across the subjects).

### The tDCS Paradigm

We adopted the same tDCS montage as that used in previous studies (Civile, Cooke, et al., 2020; Civile, McLaren, et al., 2018; Civile et al., 2019; Civile, McLaren et al., 2020; Civile, McLaren, et al., 2021; Civile, Quaglia, et al., 2021; Civile, Verbruggen, et al., 2016; Civile, Waguri, et al., 2020), based on a bilateral bipolar-non-balanced montage with one of the electrodes (anode/target) placed at Fp3 and the cathode/reference was placed on the opposite supraorbital area (on the Fp2 EEG channel, approximately above the right eyebrow).

In the anodal condition group, a direct current stimulation of 1.5 mA was delivered continuously for 10 min with 5 s fade-in and 5 s fade-out starting as soon as the subjects began the behavioural task and continuing throughout the study phase. In the sham condition group, subjects experienced the same 5 s fade-in and 5 s fade-out, but with the stimulation intensity of 1.5 mA delivered for just 30 s (Figure 2a). For every subject the stimulation started at the beginning of the study phase and ended before the recognition task started.

The tDCS stimulation was delivered by a battery driven, constant current stimulator, Neuroelectrics (https://www.neuroelectrics.com), via a pair of surface sponge electrodes (35 cm^2^), soaked in a saline solution and applied to the scalp at the target areas of stimulation. A double-blind procedure was used reliant on the Neuroelectrics system double-blind mode.

### EEG Recordings

As in Civile, Waguri, et al. (2020) EEG recordings were obtained using the Enobio system from Neuroelectrics. The control unit Necbox connects through Wi-Fi to the Neuroelectrics Instrument Controller (NIC) software running on a computer. EEG data is streamed via Wi-Fi and it is sampled at 500 SPS with a bandwidth of 0 to 125 Hz (DC coupled). The Driven Right Leg (DRL) and the Common Mode Sense (CMS) connections correspond to the electrical reference, or “ground”, of the system. The DRL is responsible for bringing the potential of the subject as close as possible to the “zero” of the electrical system. Specifically, in the Enobio 20-channel configuration used here, the CMS/DRL electrode is represented by the EarClip dual electrode system applied to the earlobe through conductive gel. In NIC, the quality of the EEG signals is assessed via the quality index (QI) which is computed every 2 s and is dependent on the following parameters: (i) Line Noise power (μV2) of the signal in the standard line noise frequency band (50 ± 1 Hz); (ii) Main noise signal power of the standard EEG band (1–40 Hz); (iii) Offset, mean value of the waveform; (iv) Drift, which is measured but not included in the QI computation because it has a high inter-subject variability. Before starting the recording (and the tDCS stimulation), the experimenter ensured that the QI for each channel was indicated as “good.”

### EEG Data Processing and N170 Analysis

EEG data were processed following the procedure adopted in Civile, Zhao et al. (2014, Experiment 4), Civile, Cooke et al. (2020, Experiment 1) and Civile, Waguri et al. (2020, Experiment 1). We used MATLAB with the open-source EEGLAB (Delorme & Makeig, 2004) and ERPLAB (Lopez-Calderon & Luck, 2014) toolboxes. Data were filtered off-line using a noncausal Butterworth bandpass filter (half-amplitude cut-offs at 0.1 and 20 Hz, 24 dB/octave roll-off). All scalp electrodes were referenced off-line to a Cz reference. Bad parts of the EEG recording were identified and removed using EEGLab's *pop_rejcont* function. To correct for blink artefacts, independent component analysis was applied to the continuous data after the deletion of sections containing extreme values. Artefact-free data were then segmented into epochs ranging from 250 ms before to 800 ms after stimulus onset for all conditions (Zion-Golumbic & Bentin, 2007).

ERPs were created by averaging the segmented trials (and baseline corrected using the mean voltage of the 100 ms pre-stimulus) according to the stimulus’ conditions in the study phase and recognition phase. The absolute peak of the N170 was determined using the ERPLAB Measurement Tool based on the option to select the most negative peaks between 130 and 220 ms. Subsequent visual scrutiny was applied to ensure that the values represented real peaks rather than end points of the epoch. The ERP N170 latency and amplitude analyses were restricted to electrode PO8 over the right temporal hemisphere, which often in the literature has shown bigger effects on the N170 in response to face stimuli (Civile, Cooke, et al., 2020; Civile, Elchlepp, et al., 2018; Civile, Waguri, et al., 2020; Civile, Zhao, et al., 2014; Prieto et al., 2011; Rossion & Jacques, 2008; Navajas et al., 2013).

## Results

### Behavioural Data Analysis

The primary measure was the accuracy data from all subjects for the normal faces. These were used to compute a *d’* sensitivity measure (Stanislaw & Todorov, 1999) for the old/new recognition task (seen and unseen stimuli for each stimulus type) where a *d’* of 0 indicates chance-level performance. To calculate *d’*, we used subjects’ hit rate (H), the proportion of SEEN (i.e., “old”) trials to which the participant responded SEEN, and false alarm rate (F), the proportion of Not SEEN (i.e., “new”) trials to which the participant responded SEEN. Each *p*-value reported for the comparisons between conditions is two-tailed, and we also report the *F* or *t* value along with effect size (η^2^_p_). We assessed performance against chance to show that normal faces in both the tDCS sham and anodal groups were recognized significantly above chance (For all conditions we found *p* < .001 for this analysis). For completeness we also conducted the statistical analyses for the behavioural and EEG data in response to the Thatcherized faces which revealed no effect of *Face Orientation* (upright, inverted) nor of the *tDCS stimulation* (sham or anodal) and no significant interaction. We also conducted the statistical analyses for the raw accuracy data for both normal and Thatcherized faces which confirmed what we found from the d-prime analyses. Finally, we conducted Bayes factor analyses using the procedure outlined by Dienes (2011), assuming a one-tailed distribution for our theory and a mean of 0. We report the results from these extra analyses in the Supplemental Material file.

We computed a 2 × 2 mixed model design using, as a within-subjects factor, *Face Orientation* (normal upright or normal inverted) and between-subjects factor *tDCS Stimulation* (sham or anodal). Analysis of variance (ANOVA) revealed a significant main effect of *Face Orientation F*(1, 70) = 40.36, *p* < .001, η^2^_p _= .36 with upright faces (M = 1.06, SD = .51) being recognized better than inverted ones (M = .38, SD = .40), that is, the expected face inversion effect. A significant interaction *Face Orientation* × *tDCS Stimulation* was found, *F*(1, 70) = 5.26, *p* = .025, η^2^_p _= .07. No significant main effect of the between-subjects factor *tDCS Stimulation* was found, supporting the fact that the tDCS does not simply affect overall performance, *F*(1, 70) = .871, *p *= .35, η^2^_p _= .01. Hence, in agreement with Civile, Cooke, et al. (2020)'s findings, we found an enhanced face inversion effect in the anodal group with performance for upright faces (M = 1.25, SD = .72) being significantly higher than that for inverted faces (M = .33, SD = .12), *t*(35) = 5.94, *p* < .001, η^2^_p _= .50. A significant but smaller standard inversion effect was found in the sham group with performance for upright faces (M = 0.87, SD = .14) being significantly higher than that for inverted faces (M = .44, SD = .11), *t*(35) = 2.96, *p* = .006, η^2^_p _= .20. In agreement with Civile, Cooke, et al. (2020), we conducted an additional analysis to directly compare the performance for upright faces in the sham group versus that in the anodal group. Our results confirmed that the anodal tDCS manipulation has improved recognition performance for upright faces, *t*(70) = 2.02, *p* = .047, η^2^_p _= .05. No significant difference was found between performance for inverted faces in the sham versus anodal condition, *t*(70) = .641, *p* = .52, η^2^_p _< .01 (Figure 2c).

### N170 Peak Latency Analysis

We conducted a 2 × 2 × 2 ANOVA using, as a within-subjects factor, *Face Orientation* (normal upright or normal inverted), *Experiment Phase* (study phase or Recognition) and between-subjects factor *tDCS Stimulation* (sham or anodal). ANOVA revealed no significant main effect of *Experiment Phase F*(1, 70) = 1.06, *p* = .31, η^2^_p _= .01, nor the interaction *Experiment Phase* × *tDCS Stimulation*, *F*(1, 70) = .475, *p* = .49, η^2^_p _< .01, nor the interaction *Experiment Phase* × *Face Orientation*, *F*(1, 70) = 2.97, *p* = .09, η^2^_p _= .04. No significant three-way interaction (*Face Orientation × Experiment Phase × tDCS Stimulation*) was found, *F*(1, 70) = .023, *p* = .88, η^2^_p _< .01.

We found a significant main effect of *Face Orientation*, *F*(1, 70) = 7.29, *p* = .009, η^2^_p _= .09, with inverted faces eliciting a larger N170 (M = 173 ms, SD = 13.25) compared to that elicited by upright faces (M = 168.5 ms, SD = 11.08), that is, the inversion effect on the N170 latency, importantly the interaction *Face Orientation × tDCS Stimulation* was significant, *F*(1, 70) = 4.70, *p* = .034, η^2^_p _= .06. No significant main effect of *tDCS Stimulation* was found, *F*(1, 70) = .232, *p* = .63, η^2^_p _< .01. In agreement with Civile, Waguri, et al. (2020), a significant inversion effect on the N170 latency was found in the sham group where normal inverted faces (M = 175 ms, SD = 20.69) elicited a delayed N170 versus that elicited by upright faces (M = 168 ms, SD = 17.80), *t*(35) = 3.47, *p* < .001, η^2^_p _= .26. Critically, in the anodal group, the inversion effect on the N170 was not significant, with normal inverted faces (M = 170 ms, SD = 19.79) eliciting a similar N170 latency to that for the upright faces (M = 169 ms, SD = 20.31), *t*(35) = .373, *p* = .71, η^2^_p _< .01. No difference was found between the N170 latency for upright stimuli in the sham versus anodal group, *t*(70) = .155, *p* = .87, η^2^_p _< .01. Despite a numerically delayed N170 for the inverted faces in the sham compared to the anodal group, no significant difference was found here either, *t*(70) = .309, *p* = .75, η^2^_p _< .01.

### N170 Peak Amplitude Analysis

ANOVA revealed no significant main effect of *Experiment Phase F*(1, 70) = .040, *p* = .84, η^2^_p _< .01, nor the interaction *Experiment Phase* × *tDCS Stimulation*, *F*(1, 70) = .178, *p* = .67, η^2^_p _< .01, nor the interaction *Experiment Phase* × *Face Orientation*, *F*(1, 70) = 2.30, *p* = .13, η^2^_p _= .03. No significant three-way interaction (*Face Orientation × Experiment Phase × tDCS Stimulation*) was found, *F*(1, 70) = 2.87, *p* = .095, η^2^_p _= .04.

**Figure 3.**
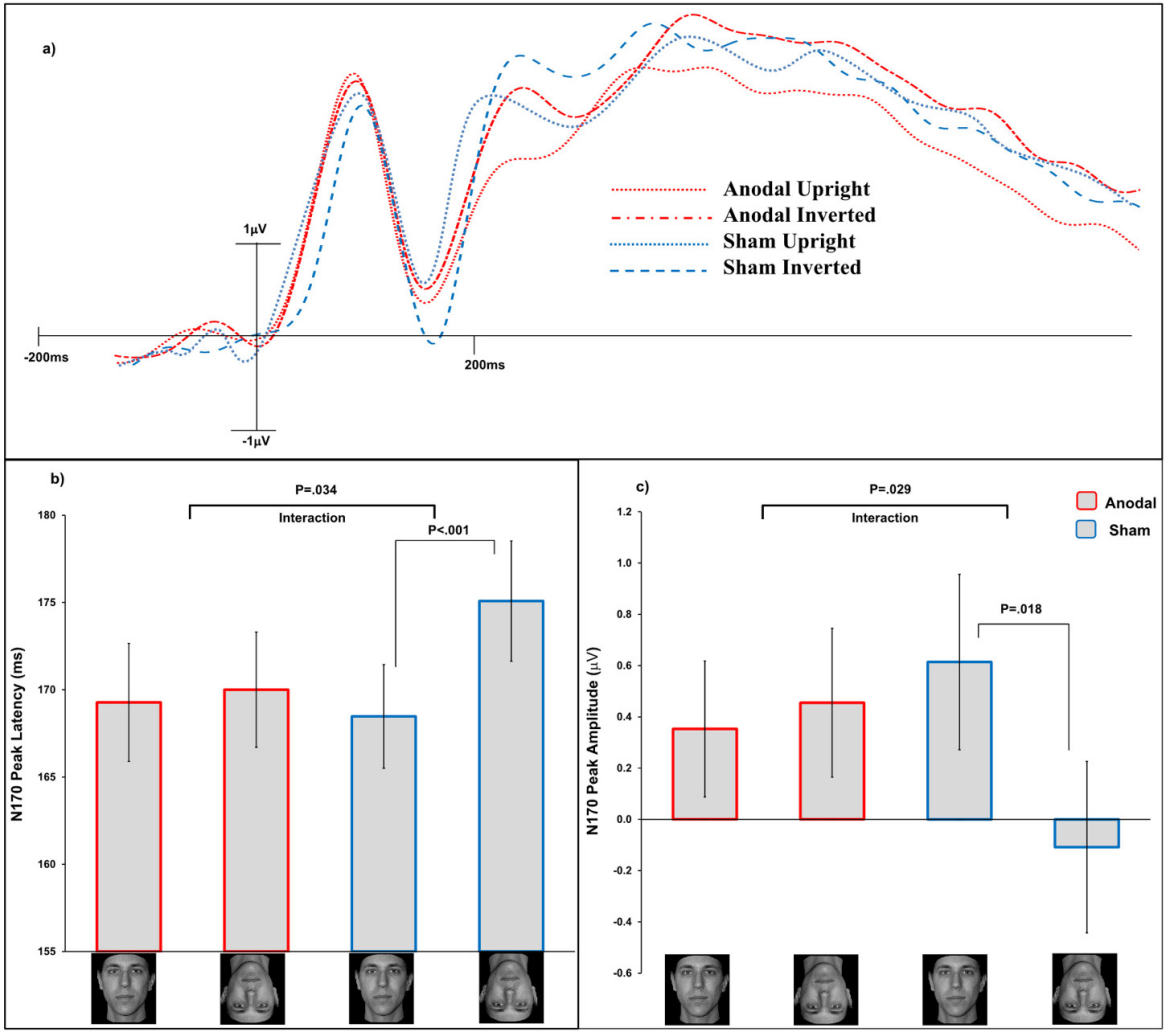
*Panel a*, waveforms at electrode P08 for the normal faces across the two tDCS conditions. The *x*-axis shows the elapsed time after a stimulus was presented. The *y*-axis gives the amplitudes (μV) of the ERPs in the study phase and recognition phase averaged together. *Panel b*, mean peak latencies (ms) for the N170 component in all conditions. *Panel c*, mean peak amplitudes (μV) for the N170 component in all conditions. Error bars are s.e.m.

We found no significant main effect of *Face Orientation*, *F*(1, 70) = 2.80, *p* = .098, η^2^_p _= .04, nor significant main effect of *tDCS Stimulation*, *F*(1, 70) = .145, *p* = .70, η^2^_p _< .01. Importantly, the interaction *Face Orientation × tDCS Stimulation* was significant, *F*(1, 70) = 4.95, *p* = .029, η^2^_p _= .06. A significant inversion effect on the N170 amplitude was found in the sham group where normal inverted faces (M = −.108 μV, SD = 2.01) elicited a larger N170 vs. that elicited by upright faces (M = .614 μV, SD = 2.05), *t*(35) = 2.48, *p* = .018, η^2^_p _= .15. Critically, in the anodal group, the inversion effect on the N170 amplitude was not significant, with normal inverted faces (M = .455 μV, SD = 1.74) eliciting a similar N170 amplitude to that for the upright faces (M = .354 ms, SD = 1.59), *t*(35) = .446, *p* = .65, η^2^_p _< .01. No difference was found between the N170 amplitude for upright stimuli in the sham versus anodal group, *t*(70) = .705, *p* = .48, η^2^_p _< .01, nor for inverted faces in the sham compared to the anodal group, *t*(70) = .386, *p* = .70, η^2^_p _< .01 (Figure 3).

## Discussion

Previous research has shown how a specific tDCS procedure can selectively modulate the inversion effect for checkerboard and face stimuli (Civile, Quaglia, et al., 2021). It has been established that anodal tDCS delivered at Fp3 (for 10 min at 1.5 mA intensity) could reduce the inversion effect for normal faces when they are the only stimulus category presented, compared to a sham tDCS group. The reduction of the inversion effect in the anodal group was shown to be mainly due to anodal tDCS affecting performance for upright faces, essentially making recognition worse for these faces than sham. And it has also been demonstrated that when the same anodal tDCS procedure was applied in circumstances where normal faces were intermixed with Thatcherized faces, the inversion effect for normal faces was increased compared to sham. In this case, performance for upright normal faces was enhanced compared to sham, so tDCS had improved recognition performance (Civile, Cooke, et al., 2020; see Civile et al., 2023 for simulations of these effects).

A recent study combined tDCS and EEG to characterize the neural signature of the tDCS effects on the inversion effect for normal faces when presented alone (i.e., not intermixed with other stimuli). The behavioural results confirmed that anodal tDCS at Fp3 (with the cathode/reference electrode placed at Fp2) reduces the inversion effect for normal faces presented alone by means of impaired recognition for upright faces. The ERP results on the N170 latencies revealed a reduced inversion effect in the anodal compared to the sham condition. In contrast the results on the N170 amplitude showed an increased inversion effect in the anodal condition compared to sham (Civile, Waguri, et al., 2020). The authors suggested that the anodal tDCS effects on the behavioural and N170 latency inversion effect may in some way reflect the disruption of perceptual learning induced by the tDCS procedure (Civile, Waguri, et al., 2020). We noted that the results on the N170 amplitudes were similar to the results obtained from studies where fixations were enforced on the eye regions, or the eyes were presented in isolation (Itier et al., 2006; Itier et al., 2007; Nemrodov et al., 2014), so that one possible mechanism producing this result would be a focus more on the facial features rather than on the configural information typically used in face recognition (i.e., spatial relationships among the main facial features; see Maurer et al., 2002 for a review on configural processing and face recognition).

In this paper, we extend Civile, Waguri, et al. (2020)'s work by applying the tDCS and EEG in combination to the task used by Civile, Cooke, et al. (2020) where normal and Thatcherized faces are intermixed. We did this because we could predict that behaviourally tDCS in these circumstances would result in an increase in the inversion effect for normal faces (see Civile et al., 2023 for simulations work), and this would allow us to see if the effects on the N170 latency and amplitude were correlated with behavioural performance or dissociated from it.

The behavioural results from our study confirmed what has been previously found in the literature. In the anodal condition the inversion effect for normal faces was significantly larger compared to the one found in the sham condition. And the Bayesian analysis confirmed that our results are in line with previous work. Critically, recognition performance for normal upright faces was better in the anodal condition compared to that in the sham condition providing some evidence for the beneficial effect of the anodal tDCS delivered at Fp3 area when normal faces are presented with Thatcherized ones. In agreement with previous studies, we found no significant differences between recognition performance for inverted faces in the anodal versus sham condition and we found no main effect of tDCS supporting the fact that this procedure does not simply reduce or enhance overall performance. Behaviourally, the tDCS procedure would affect upright faces more based on the fact that these are the stimuli for which we have developed more perceptual learning because we do not have as much expertise at seeing faces inverted upside down (Civile, Cooke, et al., 2020; Civile, McLaren, et al., 2018; Civile et al., 2019; Civile, McLaren et al., 2020; Civile, McLaren, et al., 2021; Civile, Quaglia, et al., 2021; Civile, Waguri, et al., 2020). Thus, based on the MKM model's simulations work (Civile et al., 2023) and previous tDCS studies (Civile, Cooke, et al., 2020), the tDCS procedure when applied in circumstances where normal faces are intermixed with Thatcherized faces can remove the harmful generalization from Thatcherized faces onto the normal ones.

The results from the ERPs reveal a reduced inversion effect for normal faces on the N170 latencies in the anodal versus sham condition. This is in line with previous findings (as also supported by the Bayes factor analysis) in Civile, Waguri, et al. (2020) where the same tDCS/EEG technique was applied to an old/new recognition task involving normal faces only. However, in contrast with what is found in Civile, Waguri, et al. (2020), in our study the N170 amplitudes gave reduced inversion effect for normal faces in the anodal versus sham condition. The Bayes factor analysis that we conducted provided further support to the claim that anodal stimulation reduces the inversion effect for normal faces on the N170 amplitudes. Furthermore, in the anodal condition the reduced inversion effect for normal faces on the N170 latency did not match with the behavioural enhanced face inversion effect for the same stimuli. This suggests that whether the behavioural inversion effect for normal faces is enhanced or reduced by anodal tDCS of the type we use (Civile, Waguri, et al., 2020), this form of neurostimulation would always reduce the inversion effect on the N170 latency, making it a neural signature of the effect that our procedure has.

A potential explanation for the reduced inversion effect for normal faces on the N170 latency would be our hypothesized change in feature salience modulation. If we adopt this explanation, we will argue that anodal stimulation at Fp3 affects feature salience modulation, and in circumstances where normal faces are presented alone this leads to increased generalization that leads to reduced discriminability among the faces resulting in a reduced inversion effect (Civile, McLaren, et al., 2018; Civile et al., 2019). However, when normal faces are presented with Thatcherized faces, the downregulation of feature salience modulation can help performance by removing the harmful generalization from the Thatcherized faces to the normal ones (Civile, Cooke, et al., 2020). This would lead to an enhanced behavioural inversion effect for normal faces, even though the effect of tDCS (and hence the effect on the N170 latency) is the same as before.

However, it is important to notice that whereas behaviourally the reduction (Civile, Waguri, et al., 2020) or increase (our current study) of the inversion effect for normal faces is mainly due to a modulated performance for upright faces in the anodal group, no significant difference is found between anodal versus sham upright faces on the N170 latency. This discrepancy between behavioural results and those on the N170 component has been found before in studies investigating the inversion effect when performance for upright and inverted faces was examined in isolation. For instance, Civile, Elchlepp, et al. (2018) used scrambled faces to manipulate the inversion effect. The behavioural results revealed how the inversion effect was significantly reduced compared to that for normal faces mainly due to a disrupted performance for upright scrambled faces. The EEG data also revealed that the inversion effect on the N170 latency was significantly reduced scrambled versus normal faces however no significant difference was found between upright normal versus scrambled faces. Similar discrepancies between behavioural and N170 effects for upright faces taken in isolation were also found in Civile, Cooke, et al. (2020) using normal versus Thatcherized faces, Civile, Zhao, et al. (2014) using familiar upright versus novel upright checkerboards and by Vizioli et al. (2010) using other race faces.

Coming back to our study, in contrast to Civile, Waguri, et al. (2020)'s findings, our results revealed a reduced inversion effect on the N170 amplitude in the anodal condition compared to that found in the sham condition. In some sense, then, the effect on the N170 amplitude correlates with behavioural performance. In Civile, Waguri, et al. (2020), the same tDCS procedure reduced the behavioural inversion effect and increased the difference in N170 amplitudes for upright and inverted faces. Now, when tDCS increases the behavioural inversion effect, the difference in amplitudes is decreased. It would seem that the two effects are negatively correlated. A potential explanation for this result is based on the specific manipulation used in our study to influence generalization (i.e., adding the Thatcherized faces). According to the perceptual learning account considered here and used to explain the results of previous studies, the tDCS procedure can increase generalization when normal faces are presented alone and reduce generalization when normal faces are presented with Thatcherized ones (Civile, Cooke, et al., 2020). The inversion effect for normal faces on the N170 amplitude is positively correlated with this hypothesized change in generalization.

Another (somewhat related) potential explanation for the effects obtained on the N170 amplitudes in our current study is based on previous work by Jacques et al. (2007) that used a similar behavioural paradigm to ours. The authors found that if you presented an upright face for 3 s, and then followed it shortly afterwards (300 msec) with another upright face, then the first presentation affects the N170 to the second. If the first face was the same as the second, then the N170 was smaller, in fact, a smaller amplitude was the main effect, latency was relatively unchanged. If they were different faces, then the amplitude was larger. There was much less of an effect for inverted faces. The implications of this for Civile, Waguri, et al. (2020)'s studies are that the similarity of one face to another might affect the N170 amplitude for upright faces. Now if this is the case, then if we increase that similarity then the amplitude for upright faces should decrease, increasing the difference between upright and inverted faces. That would explain the amplitude difference increase in Civile, Waguri, et al. (2020), and the increased similarity between the upright faces would degrade performance to the upright faces reducing the behavioural inversion effect. On the other hand, intermixing normal and Thatcherized faces as in this paper, increases generalization between the faces which equates to more similarity and hence should also increase the amplitude difference in the sham group. When the tDCS procedure is applied, the Thatcherized faces change from being more “similar” (in the sense of more generalization) to normal faces than other normal faces to being much less similar (in the sense of reduced generalization). This leads to a reduction in the amplitude difference accompanied by an increase in the behavioural inversion effect. At this stage, further studies should investigate this directly perhaps by extending the tDCS/EEG procedure to the inversion effect for normal faces only paradigm and that for normal faces presented with Thatcherized faces paradigm within the same study. This would allow a direct comparison of the N170 modulations. A similar study was conducted by Civile, Cooke, et al. (2020) to compare within the same study the tDCS-induced effects on the inversion effect, however no EEG recording was possible at the time.

To sum up, we found that the inversion effect for normal faces on the ERP N170 component is modulated by tDCS in a complex fashion. The effect on amplitudes tracks the behavioural effect (albeit they are negatively correlated), whilst the effect on latencies seems to be invariant with respect to the impact on behaviour. This dissociation of these different components of the N170 is the main result of this paper and constrains theories of the N170 and the neural mechanisms that generate it. This study is the first to look at the N170 correlates of the tDCS effects on the inversion effect for normal faces when intermixed with Thatcherized faces. The results advance our understanding of how a specific tDCS procedure can systematically enhance face recognition performance (measured through the size of the inversion effect) and thus modulate the electrophysiological correlates of perceptual learning and face recognition.

More generally, our results contribute to a recent line of research investigating the effects of tDCS on face recognition, though some of these studies tend to differ in detail from our procedure. For instance, Pisoni et al. (2015) investigated the effects of tDCS on a face-name association learning task showing how anodal stimulation delivered over the left anterior temporal lobe at T3 location disrupted performance compared to sham. Barbieri et al. (2016) provided evidence of how anodal tDCS at PO8 can enhance face and object recognition performance. Yang et al. (2014) showed how anodal tDCS at P8 can modulate face recognition performance as indexed by the size of the composite face effect, however, the authors provided no further analyses to reveal if the effects were due to an enhanced or reduced performance for any of the conditions used. Contrarily, Renzi et al. (2015) found that anodal tDCS at OFA area did not influence the composite face effect (similarly to Civile, McLaren, et al., 2021). However, when the authors extended the same tDCS procedure to a Mooney Task (black and white distorted images), a blocking learning effect was found at face detection and a decreased performance at object detection (Renzi et al., 2015). More recently, Civile et al. (2022) showed that anodal tDCS delivered at Fp3 (same as our procedure) influenced subjects’ decision criterion when performing a face target detection task. Interestingly, two recent studies have also investigated the effects of tDCS on the other-race effect (ORE). Costantino et al. (2017) showed that cathodal tDCS delivered at PO8 can induce ORE-like effects (i.e., reduced face recognition performance) in non-Western Caucasian subjects when asked to recognize Western Caucasian faces. Civile and McLaren (2022) provided the first evidence in the literature of how the ORE indexed by the face inversion effect (i.e., larger inversion effect for own vs. other-race faces) can be reduced after administering the anodal tDCS at Fp3. Hence, Western Caucasian subjects assigned to the anodal stimulation group revealed a reduced inversion effect for own-race faces down to a similar level to that obtained for other-race faces, essentially eliminating the ORE.

In conclusion, in this paper we characterized the effects of tDCS in modulating perceptual learning as indexed by the face inversion effect and the N170. Our work also contributes to the investigation of the effect of tDCS on face recognition, a recently developed research area containing studies that have demonstrated different effects depending on the task and the experimental design used.

## Supplemental Material

sj-docx-1-pec-10.1177_03010066231215909 - Supplemental material for Using transcranial direct current stimulation (tDCS) to selectively modulate the face inversion effect and N170 event-related potentials Click here for additional data file. Supplemental material, sj-docx-1-pec-10.1177_03010066231215909 for Using transcranial direct current stimulation (tDCS) to selectively modulate the face inversion effect and N170 event-related potentials by Ciro Civile, Emika Waguri and I.P.L. McLaren in Perception
