# Supplementary material for: Using transcranial direct current stimulation (tDCS) to selectively modulate the face inversion effect and N170 event-related potentials
Source: Perception. 2023 Nov 28;53(2):125–42. doi: 10.1177/03010066231215909 (PMC10798030; doi:10.1177/03010066231215909)

**Supplemental Material for:**

**Using transcranial Direct Current Stimulation (tDCS) to selectively modulate the face inversion effect and N170 Event-Related Potentials.**

Ciro Civile, Emika Waguri, and I.P.L. McLaren

**Part a) Additional Bayes factor analyses**

A Bayes analysis on the difference between the *d’* values for upright and inverted normal faces (i.e., the inversion effect score) comparing sham and anodal groups (thus capturing the 2 × 2 interaction) was run. We used as the *priors* the differences found in Civile, Cooke et al (2020)’s Experiment 2 and 3b averaged together, setting the standard deviation of p (population value | theory) to the mean for the difference between the inversion effect in sham group vs that in the anodal group (0.35). We used the standard error (0.15) and mean difference (0.49) between the inversion effect in the sham group vs that in the anodal group in our study. This gave a Bayes factor of 33.41, which is very strong evidence (greater than 10, for the conventional cut-offs) that these results are in line with previous work i.e., the tDCS procedure used here increases the face inversion effect.

We conducted a further a Bayes analysis this time on the difference between the N170 latencies for inverted and upright normal faces (i.e., the inversion effect on the N170 latency) comparing the sham and anodal groups capturing the 2 × 2 interaction. We used as the *priors* the differences found in Civile, Waguri et al (2020)’s Experiment 1 and 2 averaged together, setting the standard deviation of p (population value | theory) to the mean for the difference between the inversion effect in sham group vs that in the anodal group (7.08). We used the standard error (2.11) and mean difference (5.89) between the inversion effect on the N170 latency in the sham group vs that in the anodal group in our study. This gave a Bayes factor of 20.21, which is strong evidence that these results are in line with previous work i.e., the tDCS procedure reduces the inversion effect on the N170 latency.

We also conducted a somewhat different Bayesian analysis for the effect of anodal tDCS on the inversion effect on the N170 amplitude. The question we tried to answer is the following: Given that the effect can be as large as is found in the sham condition, is the effect in the anodal tDCS condition part of that population, or is it better described as null (mean of zero)? We used as a prior the mean difference for upright and inverted normal faces N170 amplitudes (i.e., inversion effect) in the sham group (0.72), and the standard error (0.16) and mean difference (-0.10) for the inversion effect on the N170 amplitude in the anodal group. This gave a Bayes factor of 0.03, which is less than 0.3. and hence can be considered strong evidence for the null, supporting the claim that the anodal stimulation reduces the face inversion effect on the N170 amplitudes.

**Part b) Additional Analyses on N170 for the normal faces using the Common Average**

Here we conducted the same statistical analyses as in the manuscript, but for our EEG processing we used the common average instead of Cz as reference. The results confirmed the effects obtained by using Cz as reference.

**N170 Peak Latency Analysis**. We conducted a 2 x 2 x 2 ANOVA using, as a within-subjects factor, *Face Orientation* (normal upright or normal inverted), *Experiment Phase* (study phase or Recognition) and between-subjects factor *tDCS Stimulation* (sham or anodal). Analysis of Variance (ANOVA) revealed no significant main effect of *Experiment Phase* *F*(1, 70) = 1.19, *p* = .27, η^2^_p_ = .01, nor the interaction *Experiment Phase* x *tDCS Stimulation*, *F*(1, 70) = .001, *p* = .97, η^2^_p_ < .01, nor the interaction *Experiment Phase* x *Face Orientation*, *F*(1, 70) = .011, *p* = .91, η^2^_p_ < .01. No significant three-way interaction (*Face Orientation x* *Experiment Phase* *x* *tDCS Stimulation*) was found, *F*(1, 70) = .155, *p* = .69, η^2^_p_ < .01.

We found a significant main effect of *Face Orientation*, *F*(1, 70) = 4.95, *p* = .029, η^2^_p_ = .06, with inverted faces eliciting a larger N170 (M = 166 ms, SD = 17.23 ) compared to that elicited by upright faces (M = 163 ms, SD = 16.11) i.e., the inversion effect on the N170 latency, importantly the interaction *Face Orientation x tDCS Stimulation* was significant, *F*(1, 70) = 6.27, *p* = .015, η^2^_p_ = .08. No significant main effect of *tDCS Stimulation* was found, *F*(1, 70) = .048, *p* = .82, η^2^_p_ < .01. A significant inversion effect on the N170 latency was found in the sham group where normal inverted faces (M = 167 ms, SD = 24.26) elicited a delayed N170 vs that elicited by upright faces (M = 160 ms, SD = 21.75), *t*(35) = 3.30, p = .002, η^2^_p_ = .24. Critically, in the anodal group, the inversion effect on the N170 was not significant, with normal inverted faces (M = 164 ms, SD = 24.24) eliciting a similar N170 latency to that for the upright faces (M = 165 ms, SD = 23.20), *t*(35) = .201, p = .84, η^2^_p_ < .01. No difference was found between the N170 latency for upright stimuli in the sham vs anodal group, *t*(70) = .875, p = .38, η^2^_p_ < .01, nor between the N170 for the inverted faces in the sham compared to the anodal group, *t*(70) = .403, p = .68, η^2^_p_ < .01.

**N170 Peak Amplitude Analysis**. Analysis of Variance (ANOVA) revealed no significant main effect of *Experiment Phase* *F*(1, 70) = .311, *p* = .58, η^2^_p_ < .01, nor the interaction *Experiment Phase* x *tDCS Stimulation*, *F*(1, 70) = .061, *p* = .80, η^2^_p_ < .01, nor the interaction *Experiment Phase* x *Face Orientation*, *F*(1, 70) = .189, *p* = .66, η^2^_p_ < .01. No significant three-way interaction (*Face Orientation x* *Experiment Phase* *x* *tDCS Stimulation*) was found, *F*(1, 70) = .242, *p* = .624, η^2^_p_ < .01.

We found no significant main effect of *Face Orientation*, *F*(1, 70) = 3.53, *p* = .065, η^2^_p_ = .05, nor significant main effect of *tDCS Stimulation*, *F*(1, 70) = 1.37, *p* = .25, η^2^_p_ = .02. Importantly, the interaction *Face Orientation x tDCS Stimulation* was significant, *F*(1, 70) = 5.07, *p* = .028, η^2^_p_ = .06. A significant inversion effect on the N170 amplitude was found in the sham group where normal inverted faces (M = -.894 μV, SD = 2.44) elicited a larger N170 vs that elicited by upright faces (M = -.023 μV, SD = 2.13), *t*(35) = 2.60, p = .013, η^2^_p_ = .16. Critically, in the anodal group, the inversion effect on the N170 amplitude was not significant, with normal inverted faces (M = .131 μV, SD = 2.11) eliciting a similar N170 amplitude to that for the upright faces (M = .055 μV, SD = 2.02), *t*(35) = .292, p = .77, η^2^_p_ < .01. No difference was found between the N170 amplitude for upright stimuli in the sham vs anodal group, *t*(70) = .16, p = .87, η^2^_p_ < .01 nor for inverted faces in the sham compared to the anodal group, *t*(70) = 1.9, p = .07, η^2^_p_ = 05.

**Part c) Results for the Thatcherized Faces**

**D-prime Behavioural Data Analysis.** We computed a 2 x 2 mixed model design using, as a within-subjects factor, *Face Orientation* (upright or inverted) and between-subjects factor *tDCS Stimulation* (sham or anodal). Analysis of Variance (ANOVA) revealed no significant main effect of *Face* *Orientation F*(1, 70) = .04, *p* = .84, η^2^_p_ < .01 [Thatcherized upright M = .62, SD = .63; Thatcherized inverted M = .60, SD = .66], nor significant interaction *Face Orientation* x *tDCS Stimulation* was found, *F*(1, 70) = .11, *p* = .73, η^2^_p_ < .01. No significant main effect of the between-subjects factor *tDCS Stimulation* was found, *F*(1, 70) = .015, *p* = .90, η^2^_p_ < .01.

**N170 Results (Cz as reference)**

**N170 Peak Latency Analysis**. Analysis of Variance (ANOVA) revealed no significant main effect of *Face Orientation*, *F*(1, 70) = 3.51, *p* = .067, η^2^_p_ = .04 [Thatcherized upright M = 166, SD = 19.90; Thatcherized inverted M = 168, SD = 20.75], nor interaction *Face Orientation x tDCS Stimulation*, *F*(1, 70) = 1.40, *p* = .24, η^2^_p_ = .02. No significant main effect of *tDCS Stimulation* was found, *F*(1, 70) = .099, *p* = .75, η^2^_p_ < .01.

**N170 Peak Amplitude Analysis**. Analysis of Variance (ANOVA) revealed no significant main effect of *Face Orientation*, *F*(1, 70) = .515, *p* = .475, η^2^_p_ < .01 [Thatcherized upright M = .31, SD = 1.83; Thatcherized inverted M = .48, SD = 1.88], nor *Face Orientation x tDCS Stimulation* was significant, *F*(1, 70) = .085, *p* = .771, η^2^_p_ < .01. No significant main effect of *tDCS Stimulation* was found, *F*(1, 70) = .448, *p* = .50, η^2^_p_ < .01.

**Part d) Behavioral Raw Scores Results for the Normal Faces**

We computed a 2 x 2 mixed model design using, as a within-subjects factor, *Face Orientation* (normal upright or normal inverted) and between-subjects factor *tDCS Stimulation* (sham or anodal). Analysis of Variance (ANOVA) revealed a significant main effect of *Face* *Orientation F*(1, 70) = 45.68, *p* < .001, η^2^_p_ = .39 indicating that overall upright faces (M = 10.77, SD = 1.99) were recognized better than the inverted ones (M = 8.97, SD = 1.85) i.e., the face inversion effect. A significant interaction *Face Orientation* x *tDCS Stimulation* was found, *F*(1, 70) = 5.47, *p* = .022, η^2^_p_ = .07. No significant main effect of the between-subjects factor *tDCS Stimulation* was found, supporting the fact that the tDCS does not simply affect overall performance, *F*(1, 70) = 1.61, *p* = .21, η^2^_p_ = .02. In agreement with the d-prime results we found an enhanced face inversion effect in the anodal group with performance for upright faces (M = 11.31, SD = 1.80) being significantly higher than that for inverted faces (M = 8.88, SD = 1.90), *t*(35) = 6.13, p < .001, η^2^_p_ = .52. A significant but smaller inversion effect was found in the sham group with performance for upright faces (M = 10.23, SD = 2.06) being significantly higher than that for inverted faces (M = 9.05, SD = 1.83), *t*(35) = 3.29, p = .002, η^2^_p_ = .23. We conducted an additional analysis to directly compare the performance for upright faces in the sham group vs that in the anodal group which confirmed that the anodal tDCS manipulation has improved recognition performance for upright faces, *t*(70) = 2.23, p = .032, η^2^_p_ = .14. No significant difference was found between performance for inverted faces in the sham vs anodal condition, *t*(70) = .378, p = .70, η^2^_p_ < .01 (Figure 4).

**Figure 4:** Behavioural results for the normal faces only. The x-axis represents the normal upright and inverted faces across the two tDCS conditions (anodal, sham). The y-axis represents raw scores accuracy. Error bars are s.e.m.

**Behavioral Raw Scores Results for the Thatcherized Faces**

We computed a 2 x 2 mixed model design using, as a within-subjects factor, *Face Orientation* (upright or inverted) and between-subjects factor *tDCS Stimulation* (sham or anodal). Analysis of Variance (ANOVA) revealed no significant main effect of *Face* *Orientation F*(1, 70) = .003, *p* = .96, η^2^_p_ < .01 [Thatcherized upright M = 9.57, SD = 1.63; Thatcherized inverted M = 9.56, SD = 1.69], nor significant interaction *Face Orientation* x *tDCS Stimulation* was found, *F*(1, 70) = .01, *p* = .92, η^2^_p_ < .01. No significant main effect of the between-subjects factor *tDCS Stimulation* was found, *F*(1, 70) = .283, *p* = .60, η^2^_p_ < .01.

**TABLE 1: Mean and SD Raw Scores for each stimulus’s condition**


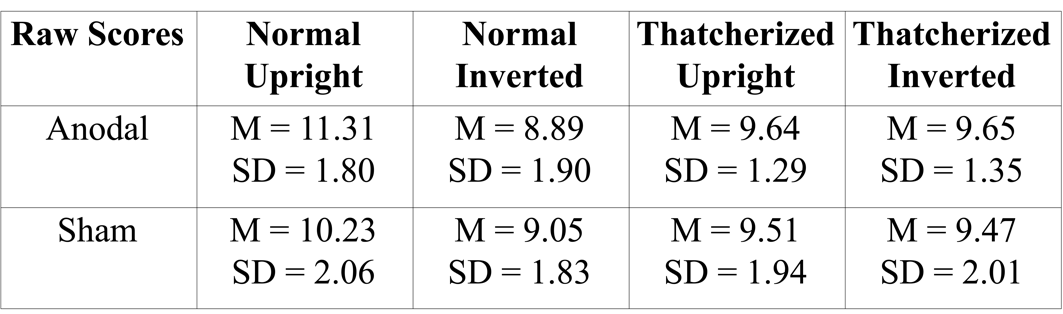

Supplement: sj-docx-1-pec-10.1177_03010066231215909 - Supplemental material for Using transcranial direct current stimulation (tDCS) to selectively modulate the face inversion effect and N170 event-related potentials [file sj-docx-1-pec-10.1177_03010066231215909.docx]
